# Insights on the Host Stress, Fear and Growth Responses to the Deoxynivalenol Feed Contaminant in Broiler Chickens

**DOI:** 10.1371/journal.pone.0087727

**Published:** 2014-01-30

**Authors:** Khaled Ghareeb, Wageha A. Awad, Omer E. Sid-Ahmed, Josef Böhm

**Affiliations:** 1 Institute of Animal Nutrition and Functional Plant Compounds, Department for Farm Animals and Veterinary Public Health, University of Veterinary Medicine, Vienna, Austria; 2 Department of Animal Hygiene, Behaviour and Management, Faculty of Veterinary Medicine, South Valley University, Qena, Egypt; 3 Clinic for Avian, Reptile and Fish Medicine, Department for Farm Animals and Veterinary Public Health, University of Veterinary Medicine, Vienna, Austria; 4 Institute of Medical Biochemistry, Department of Biomedical Sciences, University of Veterinary Medicine, Vienna, Austria; Oregon Health & Science University, United States of America

## Abstract

Mycotoxins pose an important danger to human and animal health. Poultry feeds are frequently contaminated with deoxynivalenol (DON) mycotoxin. It is thus of great importance to evaluate the effects of DON on the welfare related parameters in poultry industry. In the present study, the effects of contamination of broiler diet with 10 mg DON/kg feed on plasma corticosterone and heterophil to lymphocyte (H/L) ratio as indicators of stress, tonic immobility duration as an index for fear response and growth performance of broiler chickens were studied. In addition, the effect of a microbial feed additive either alone or in combination with DON contamination on these different aspects was also evaluated. The results showed that DON feeding significantly affected the welfare related parameters of broiler chickens. The feeding of DON contaminated diet resulted in an elevation of plasma corticosterone, higher H/L ratio and increased the fear levels as indicated by longer duration of tonic immobility reaction. Furthermore, DON reduced the body weight and body weight gain during the starter phase definitely at the second and third week. However, during grower phase, feeding of DON decreased the body weight at the fourth week and reduced the body gain at the fifth week. Addition of the microbial feed additive, a commercial antidote for DON mycotoxin, was able to overcome DON effects on stress index (H/L ratio), fearfulness and growth parameters of broilers. In conclusion, we showed for the first time that the DON feeding increased the underlying fearfulness and physiological stress responses of broilers and resulted in a reduction in the welfare status as indicated by higher plasma corticosterone, higher H/L ratio and higher fearfulness. Additionally, feeding the microbial feed additive was effective in reducing the adverse effects of DON on the bird's welfare and can improve the performance of broiler chickens.

## Introduction

Deoxynivalenol (DON), commonly known as vomitoxin, is a trichothecene mycotoxin produced by several fungal species, such as *Fusarium, Myrothecium*, *Cephalosporium*, *Verticimonosporium*, and *Stachybotrys* [1]. Cereal grains are the main constitutes of poultry feeds which are frequently contaminated with DON mycotoxin due to changes in the environmental condition such as temperature and humidity. Thus exposure of broiler chickens to DON contaminated diet can not be avoided. The impacts of DON contamination of poultry feeds on a wide range of significant health and immunological parameters were intensively studied [2], [3], [4], [5], [6], [7], [8], [9], [10], [11]. However, the effects of DON contamination of broiler diet on the welfare related parameters are not investigated, only a few studies showed that DON increased the stress index (heterophil to lymphocyte ratio, H/L ratio) in broilers [12], [13].

Stress is the loss of homeostasis by stressors. It is known that poultry in modern production have to deal with several stressors during their life such as weighing, vaccination and a lot of environmental and management procedures. These factors can affect the behavioural, physiological, and performance responses of birds. Among these factors are nutritional, climatic, social and biological conditions [14], [15], [16], [17]. These external factors are considered stressors, led to higher plasma corticosterone levels and inducing a state of stress response. Studies on the physiology of the stress indicated that corticosterone produced during acute or chronic stressor exposure is one of the final hormones of the hypothalamic-pituitary adrenal axes cascade [15], [18], having a multifunctional role by impairment of neuro-endocrine and immune functions [16]. For example, higher plasma corticosterone was responsible for many metabolic and physiological problems and modulated the immunological functions [14], [17], [19], [20] which in turn can have a significant effects on growth and health of birds during production [21].

Based on the physiological basis; the welfare of broilers can be measured using plasma corticosterone level and heterophil to lymphocyte ratio [22]. The welfare related behaviour such as fear, as measured by tonic immobility reaction, can give an indicator for stress [22], [23]. It was shown that aflatoxin (AFB1) feeding can affect the tonic immobility duration as a behavioural indicator to stress [24] in broiler chickens. Furthermore, AFB1 contamination (100 µg/kg of feed) affected the physiology of Japanese quail similar to a chronic stressor (corticosterone administration in drinking water) because it elevates the heterophil to lymphocyte ratio [25]. Similarly, AFB 1 contamination of Japanese quail feeds produced a similar effects as corticosterone administration in term of higher H/L ratio [26] and reduced the growth performance and the combination of both produced more stress. In broiler chickens feeding of 1 ppm AFB 1 resulted in higher H/L ratio which indicate a poor welfare [27].

So far, no available reports regarding the effects of DON on the plasma level of corticosterone and fearfulness as welfare related parameters in broiler chickens. The hypothesis of the present experiment is that contamination of broiler diet with 10 mg DON/kg feed can affect the welfare of broilers in the terms of plasma corticosterone level, H/L ratio, fearfulness and growth performance. From other hand, addition of a microbial feed additive (Mycofix Select) as an antidote for DON mycotoxin can remove or reduce the impacts of DON on the investigated parameters. This microbial based feed additive was capable of counteracting the immunotoxic effects induced by DON in chickens [12]. The epoxidase enzyme of *Eubacterium* sp. of Mycofix Select can metabolize DON to DOM-1 (less toxic form) by reductive cleavage of the toxic 12, 13-epoxy ring in the gut of chickens and received a positive opinion from the European Food Safety Authority (EFSA) on DON mycotoxin biotransformation. EFSA concluded that this additive is safe for animals, humans and environment and also efficacious in the target species [28]. Therefore, the present study was conducted to investigate the single and combined effects of DON and Mycofix Select on the welfare parameters such as corticosterone level, H/L ratio and tonic immobility reaction in broiler chickens.

## Materials and Methods

### Ethics statement

The animal experiments were discussed and approved by the institutional ethics committee of the University of Veterinary Medicine. All husbandry practices and euthanasia were performed with full consideration of animal welfare.

### Birds and housing

Male broiler chicks (Ross 308) were reared in battery cages from 1 d old to 35 d old. The climatic conditions and lighting program were computer-operated and followed the commercial recommendations. Environmental temperature in the first week of life was 35°C and reduced to 25°C until the end of the experiment. During the first week, 22 h of light was provided with a reduction to 20 h afterward.

### Experimental diets

Birds of each group (10) received one of the following dietary treatments; 1) basal diet; 2) basal diet contaminated with 10 mg DON/kg feed; 3) basal diet contaminated with 10 mg DON/kg feed and supplemented with a microbial feed additive, Mycofix Select (MS) (2.5 kg/ton of feed); 4) basal diet supplemented with a microbial feed additive, Mycofix Select (MS) (2.5 kg/ton of feed). The birds were fed the starter feed from 1–13 days old and grower feed from 14–35 days old. Water and feed were available *ad libitum*. The basal diet of starter feed composed of corn 55%, soya bean high protein 29%, rapeseed oil 1%, roasted soybean 6.91%, calcium carbonate 2.03%, moncalcium phosphate 1.89%, palm oil 1.88%, pumpkin seed expeller 0.72%, sodium chloride 0.48%, L-Lysine 0.34%, methionine 0.24%, Magnesium phosphate 0.13%, L-Threonine 0.12%, vitamin premix 0.16% (MIAVIT GmbH & Co. KG, Essen, Germany, each kilogram of vitamin premix contains vitamin A, 200,000 IU; vitamin D3, 80,000 IU; vitamin E, 1,600 mg; vitamin K3, 34 mg; vitamin C, 1,300 mg; vitamin B1, 35 mg; vitamin B2, 135 mg; vitamin B6, 100 mg; vitamin B12, 670 µg; nicotinic acid, 1,340 mg; calcium pantothenic acid, 235 mg; choline chloride, 8,400 mg; folic acid, 34 mg; biotin, 3,350 µg; and methionine, 30 g), and Trace element premix 0.10%. The grower basal diet composed of corn 62%, soya bean high protein 23.80%, rapeseed oil 2.00%, roasted soybean 5.53%, calcium carbonate 1.62%, moncalcium phosphate 1.71%, palm oil 1.50%, pumpkin seed expeller 0.58%, sodium chloride 0.38%, L-Lysine 0.27%, methionine 0.20%, Magnesium phosphate 0.10%, L-Threonine 0.10%, vitamin premix 0.13%, and Trace element premix 0.08%. Ten milligrams of pure deoxynivalenol/kg were added to basal feed to constitute the DON group (artificially contaminated diet). Two and half kg of Mycofix® Select (Biomin GmbH, Herzogenburg, Austria)/ton of diet were added to constitute the Mycofix group. This plus 10 mg pure DON/kg of diet constitutes DON+Mycofix group (artificially contaminated diet + Mycofix Select). Representative feed samples were taken from starter and grower diets and were analyzed by HPLC to confirm the level of DON according to the method described earlier [29]. The level of DON in the starter basal diet was 506±80 µg/kg feed. However, the level of DON was 10,509±80 µg/kg feed for the DON-contaminated starter diet. In basal grower diet, DON concentration was 611±95 µg/kg feed while in the DON contaminated grower diet; DON was 10,614±95 µg/kg feed.

### Performance parameters

Body weight (BW) of each bird was taken at the first day of the feeding experiment and then weekly. Body weight gain for each bird was measured weekly. Feed intake for each group was calculated weekly and accordingly feed conversion rate was measured.

### Fear levels (Tonic immobility reaction)

**Figure 1:**
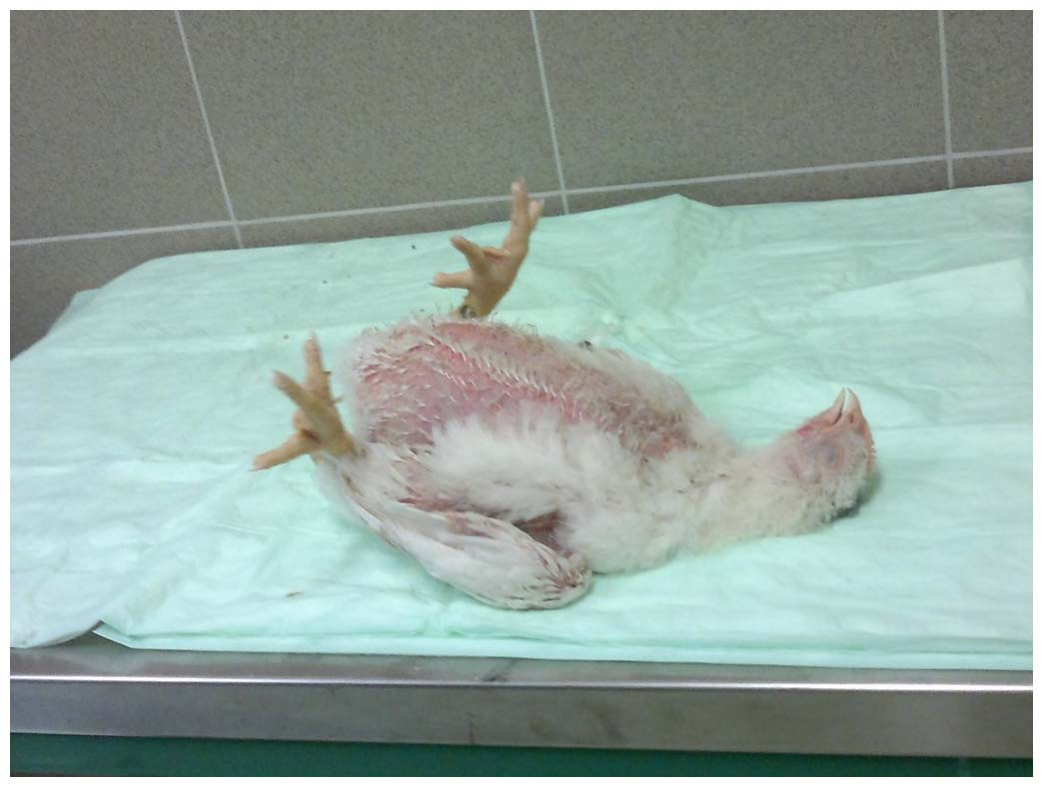
The tonic immobility reaction in broiler chickens is induced on laboratory table by gentle manual restraint. Birds of each group were subjected to the test. Both duration of tonic immobility and induction counts are recorded. The longer duration of tonic immobility indicates high fear levels. The test was performed at week 4 and week 5 of age.

A total of 40 birds (10 birds per group) were tested individually for the duration of tonic immobility reaction. This fear test was carried out according to the method described elsewhere [22], [23], [30]. Briefly, the reaction is induced by manual restraint. The individual bird was placed on its back on a laboratory table covered with cloths. The bird was then restrained with one hand on its sternum for 45 s while holding the head and neck with the other hand. The duration of tonic immobility reaction, that is, the latency until righting was recorded (Figure 1). Towards the end of the induction period, hand pressure was gradually lifted so that if the chick still moved, another induction period was started immediately, until the movement ceased. After removal of the hands, a stop watch was started. The experimenter then retreated, moving out of sight of the bird and observed the behaviour of the bird. The number of induction trials to attain tonic immobility lasting at least 20 seconds and the duration of tonic immobility reaction, that is, the latency until self righting. If the bird righted in less than 20 seconds, it was considered that tonic immobility had not been induced and the restraint procedure was repeated. Conversely, if a bird did not show a righting response over the 10 min test period, a maximum score of 600 seconds was given for duration. The fear test was performed at week 4 and 5 of age.

### Heterophil to Lymphocyte ratio

One mL of well-mixed EDTA and blood of 10 chickens per group were collected on d 35 of age for differential leukocyte counts which performed manually on 100 leukocytes by microscopic examination on Wright-Giemsa-stained blood smears to test for changes in the counts of lymphocytes and heterophils. The heterophil:lymphocyte ratio (H/L) was calculated by dividing the number of heterophils by the number of lymphocytes.

### Plasma extraction and corticosterone assaying

Blood sample from each bird was collected after slaughtering at 35 days of the feeding trial. Plasma of each bird was extracted in 13 ml glass tube with organic solvent (diethyl ether) to avoid lipid and protein interference during analysis. Briefly, 0.5 ml, was mixed with diethyl ether (10 times of the sample volume) by vortexing and centrifuged at 2500 g for ten minutes at 4°C. After separation phase, the mixture was placed at −20°C (to freeze the aqueous phase) overnight. The supernatant (diethyl ether phase) was decanted in a new clean tube and the diethyl ether was evaporated under nitrogen flow over heating block (30°C). Extracts were reconstituted with Enzyme immune-assay (EIA) buffer to their original volume (0.4 ml), vortexed and kept at −20°C until assayed. Corticosterone was assayed according to the method described by [31]. The intra and inter assay coefficient variance % (CV%) were low than 10%.

### Statistical Analysis

The statistical program SPSS (version 20; SPSS GmbH, SPSS Inc., Munich, Germany) was used for data analysis. The Kolmogorov-Smirnov test was used to test the normal distribution of the data. An ANOVA was performed between the 4 groups, followed by Duncan test to find the significance between dietary treatments. The probability values of 0.05 (P≤0.05) were considered significant.

## Results

### Performance traits

Performance traits did not show any significant differences between dietary groups at the first week of feeding trial (Table1 and 2). Feed intake of DON fed birds was numerically decreased at 4 and 5 wk (Table 1). Feed conversion rate of DON group was increased with increasing the age of birds. In contrast, feed conversion rate was decreased for Mycofix Select fed birds with increasing the age however, at wk 5 there was no difference (Table 1).

**Table 1:** Feed intake and feed conversion rate of broiler chickens fed deoxynivalenol and microbial feed additive.

Performance traits	Control group	DON group	DON + Mycofix group	Mycofix group
**WK 1:**				
Feed intake (g/bird)	182	187	171	192
Feed conversion rate	1.3	1.5	1.3	1.4
**WK 2:**				
Feed intake (g/bird)	423	431	414	430
Feed conversion rate	1.6	1.8	1.6	1.5
**WK 3:**				
Feed intake (g/bird)	601	701	666	633
Feed conversion rate	1.6	2.1	1.6	1.4
**WK 4:**				
Feed intake (g/bird)	839	676	813	819
Feed conversion rate	1.6	2.1	1.3	1.5
**WK 5:**				
Feed intake (g/bird)	792	781	676	671
Feed conversion rate	1.5	1.8	1.9	1.9

However, at 2, 3 and 4 wk, supplementation of diet with Mycofix Select resulted in higher body weight (BW) (P<0.05) compared with birds fed basal diet contaminated with DON (Table 2). As well as body weight gain (BWG) was significantly higher (P<0.05) for Mycofix group at wk 3 and 4 compared with birds fed diet contaminated with DON (Table 2).

**Table 2:** Body weight and body weight gain of broiler chickens fed deoxynivalenol and microbial feed additive.

Performance traits	Control group	DON group	DON + Mycofix group	Mycofix group	P value
**Body weight at 1 d old**	42.6±1	43±1	43±1	42.6±1	0.988
**WK 1:**					
Body weight (g)	179±4	171±6	184±8	181±7	0.574
Body weight gain (g)	136±4	128±6	141±7	139±7	0.521
**WK 2:**					
Body weight (g)	445^ab^±14	409^b^±19	434^ab^±18	462^a^±12	0.030
Body weight gain (g)	265^ab^±15	238^b^±14	250^ab^±14	281^a^±11	0.033
**WK 3:**					
Body weight (g)	810^ab^±49	746^b^±53	840^ab^±50	925^a^±27	0.009
Body weight gain (g)	365^ab^±45	336^b^±44	406^ab^±39	464^a^±21	0.024
**WK 4:**					
Body weight (g)	1330^ab^±75	1302^b^±56	1456^ab^±47	1472^a^±34	0.031
Body weight gain (g)	521^b^±32	557^b^±42	616^a^±37	547^ab^±13	0.050
**WK 5:**					
Body weight (g)	1851±109	1748±68	1798±58	1820±67	0.866
Body weight gain (g)	521^a^±45	446^b^±36	342^b^±35	348^b^±33	0.043

a,b Means within the same row with different superscripts are significantly different (ANOVA followed by Duncan test; n =  10/treatment).

Moreover, DON decreased the body weight at 1, 2, 3, and 4 and 5 week but differences did not reach significance. BW gain at end of the experiment (at week 5) was significantly decreased (P<0.05) for birds fed DON (Table 2). However at 1, 2 and 3 week the DON decreasing effect did not reach significance (Table 2). The daily body weight gain did not differ (P<0.05) between dietary treatments (Table 2).

### Fear levels

Birds fed DON showed longer tonic immobility duration and higher inductions (P<0.05) suggesting that feeding of DON elevated the fear level of birds (Table 3). Interestingly, addition Mycofix Select to DON contaminated diet decreased (P<0.05) the tonic immobility duration and its induction counts to values that are comparable to control birds (Table 3). Moreover, Mycofix Select alone decreased (P<0.05) the duration and induction counts of tonic immobility reaction compared with control (Table 3).

**Table 3:** Fear behaviour of broiler chickens fed deoxynivalenol and microbial feed additive.

Item	Control group	DON group	DON + Mycofix group	Mycofix group	P value
**Tonic immobility duration (s)**					
week 4	86^b^±30	282^a^±47	99^b^±2	185^b^±32	0.002
week 5	150^a^±24	238^b^±53	131^a^±27	142^a^±36	0.063
**Number of inductions**					
week 4	1.33^b^±0.23	1.60^a^±0.16	1.10^b^±0.10	1.20^b^±0.20	0.073
week 5	1.13±0.13	1.38±0.18	1.14±0.14	1.25±0.16	0.652

a,b Means within the same row with different superscripts are significantly different (ANOVA followed by Duncan test; n =  10/treatment).

### Stress index (H/L ratio) and plasma corticosterone

Heterophils are increased (P<0.05) and lymphocytes are decreased (P<0.05) due to DON feeding (Table 4). Moreover, H/L ratio is increased (P<0.05) due to DON exposure. Addition of a microbial feed additive reversed the adverse effects of DON on stress index by decreasing (P<0.05) heterophils and H/L ratio and increasing (P<0.05) lymphocytes compared with DON group and values of stress index return to values comparable to control values (Table 4).

**Table 4:** Stress hormone (corticosterone) and stress index (Heterophil:Lymphocyte ratio) of broilers fed deoxynivalenol and microbial feed additive.

Stress parameters	Control group	DON group	DON + Mycofix group	Mycofix group	P value
Corticosterone (ng/ml)	0.52^b^±0.2	1.53^a^±0.7	1.25^a^±0.4	0.54^b^±0.2	0.001
Heterophil %	63^b^±1	69^a^±2	62^b^±2	64^b^±2	0.035
Lymphocyte %	27^a^±2	23^b^±4	27^a^±1	27^a^±1	0.090
H/L ratio	2.39^b^±0.20	3.14^a^±0.23	2.43^b^±0.15	2.40^b^±162	0.034

a,b Means within the same row with different superscripts are significantly different (ANOVA followed by Duncan test; n =  10/treatment).

Plasma corticosterone concentration was increased (P<0.05) when birds fed DON (Table 4). However, Mycofix Select did not affect (P>0.05) plasma corticosterone level. Furthermore, feeding of DON with Mycofix Select increased (P<0.05) the plasma corticosterone concentration.

## Discussion

One of the major problems of poultry production worldwide is the threat of DON mycotoxin that is frequently detected in poultry feedstuffs [32], [33], [34]. The problem of DON contamination can be complicated because poultry are considered to be tolerant to DON toxicity compared with other species of animals and therefore the contaminated cereal grains can be directed sometimes to poultry feed production [35], [36]. Although DON mycotoxicosis is rarely happened in modern poultry industry, moderate and low DON contamination of chicken diets reduced the production and impaired the health and immunological functions [9], [36]. In chickens, DON was shown to suppress the vaccination response to infectious bronchitis virus (IBV) [2], [11], [12] and to Newcastle disease virus (NDV) [2] which indicates that DON altered the chicken humoral immunity. It was shown that DON induces chronic stress in broiler chickens as measured by H/L ratio (Stress index) due to prolonged ingestion [12], [13]. Therefore, blends of feed ingredients contaminated with DON should not be fed to broiler chickens because of the possible adverse effects on performance, immune status, and stress index. Contamination of broiler diet with DON can be considered as one of the important chronic dietary risk factor that can affects the welfare of birds. However, there are a no available reports about the effects of chronic dietary DON exposure on plasma corticosterone (stress hormone) level and fear behaviour (stress related behaviour) in broiler chickens.

Furthermore, corticosterone, the primary glucocorticoid secreted by the avian adrenal gland and reported to be elevated during stress [37], [38], induced immunosuppression and the metabolic and behavioural changes associated with stress [30]. Therefore, an array of established physiological, behavioural and performance parameters were used in this study as indicators of stress to address the impacts of DON and its antidote on the welfare of broilers. These included H/L ratios, circulating corticosterone concentrations [39], and tonic immobility duration [40], along with the performance traits [41].

The data of the present experiment showed new information about the impacts of chronic ingestion of DON for 5 weeks on the welfare of broiler chickens. The plasma corticosterone level was elevated due to DON ingestion for long period suggesting that DON produced a strong stress for broiler chickens and adversely affected the welfare of broilers. As a consequence to prolonged stress, the immune system of the birds can be affected [42]. In this respect, DON was shown to affect the humoral immune response to common vaccines [11], [12]. Indeed, the innate immune response is also altered due to chronic DON feeding. In the present trial, it was found that DON decreased the production of interleukin 8 (IL-8) which mediates the inflammatory immune response, suggesting that DON ingestion could impair immune function and elevate susceptibility to disease [43]. DON also decreased the plasma level of tumor necrosis factor alpha (TNF-α) which is a cytokine involved in systemic inflammation and stimulation of the acute phase reaction and decreased the vaccinal immune response. Furthermore, in the intestines, DON down regulated the immune response genes such as interleukin 1 beta, transforming growth factor beta receptor I and IFN gamma (IL-1β, TGFBR1, IFN-γ) [43]. Additionally, in another study, Toll-like receptors (TLR) in the intestinal epithelium, particularly TLR4 which serve as rapid pathogen sensors was up-regulated suggesting inflammed intestines [36]. In this scenario, the impairment of the immune functions of broilers could be consequences of stress induced by chronic ingestion of DON. Stress conditions that caused prolonged and frequent elevation of corticosterone in the plasma resulted in a reduction in the circulating lymphocytes, thymus involution, and decrease in the mass of spleen and peripheral lymph nodes and a reduction in immune competence [44]. Furthermore, interleukin 2 (IL-2) which activate the immune response by promoting the division of T cells and the release of the immune mediators such as interferon (INF), is decreased during stress [44]. The link between stress and immunological functions is not a new topic and widely discussed. For example, chronic stress strongly suppressed humoral and cellular immune responses as evidenced by lower antibody titers to sheep red blood cells and tetanus toxoid [30]. H/L ratio was elevated during stress and was positively correlated to higher level of corticosterone in the plasma which related to decrease the resistance to infectious diseases [39]. To our knowledge, it is the first time to address the effects of chronic stress caused by prolonged dietary DON in broiler chickens. Interestingly, addition of Mycofix Select to DON contaminated diet slightly reduced but not significantly the plasma corticosterone level. However, Mycofix addition to DON uncontaminated feed did not alter the plasma corticosterone level compared with controls.

Poultry welfare is based on health, production, behaviour, and physiology [22], [45]. Stress includes an elevation in the circulating levels of corticosterone, impairment of humoral immunity, alteration in the number of circulating leucocytes, with concomitant reduction in disease resistance, and a reduction in growth production traits [46]. Heterophils to lymphocytes ratio (H/L ratio) was concluded as a useful indicator of stress in poultry [39] and correlated positively to corticosterone level in the plasma. In the present study, the feeding of DON-contaminated diets resulted in an increase in the heterophil counts, reduction in the lymphocyte counts and higher H/L ratio compared with the controls. These responses indicate the predictable physiological changes to DON mycotoxin. Such data suggest that chronic ingestion of DON provoke a significant stress response. However, addition of Mycofix Select to DON contaminated feed was able to remove the DON effects on stress index (H/L ratio). Mycofix Select supplementation had no adverse effects on underlying fearfulness, stress reaction and performance.

Fear behaviour as indicated by the duration of tonic immobility [47] was positively correlated to H/L ratio [48], [49] and plasma corticosterone level [30], [47], [50], [51], [52], [53], [54] and consequently fear is contribute to overall stress and is used as behavioural indicator of poultry welfare [22], [30]. In the current experiment, DON ingestion resulted in prolonged duration of tonic immobility at week 4 and 5. These results suggest that DON feeding increases the fear levels of broiler chickens indicating that DON contamination of broiler diet adversely affected the welfare indicator of chickens. In addition, Mycofix Select as antidote for DON was capable to counteract the increasing level of fear produced by DON feeding. Furthermore, this feed additive did not affect the fear levels of broiler when added to the non-contaminated diet.

Performance and growth parameters were also affected by dietary DON in the present study. DON reduced the body weight and body weight gain during the starter phase (second and third week) and during grower phase, feeding of DON decreased the body weight at the fourth week and reduced the body gain at the fifth week. These results may indicate that DON decreases the welfare of broilers in the terms of affecting the growth and performance traits as a result of stress caused by DON. Additionally, Mycofix Select was able to counteract the alteration in performance trait caused by DON and can improve the performance of broiler chickens.

In conclusion, for the first time, we showed that ingestion of DON adversely affected the welfare of broiler chickens in the terms of higher plasma corticosterone level, elevated H/L ratio, higher fear levels and decreasing the body weight and body weight gain during starter and grower periods. In addition, the efficacy of Mycofix Select as a commercially antidote for DON mycotoxin was capable of removing the adverse effects of DON on the welfare of broiler chickens. Furthermore, the present results confirm that the exposure to DON can suppress the immune functions, consequently increase susceptibility to infectious diseases and contribute to the predisposition of inflammatory diseases in humans and animals, as also suggested by others [55], [56], [57], [58]. These effects of DON may be of importance for the other animal sensitive species and for public health after consuming contaminated food/feed. It is hoped that this research will increase the awareness of agriculturists and animal scientists for the important implications of feed contaminant DON on livestock welfare.
